# Differential development of neuronal physiological responsiveness in two human neural stem cell lines

**DOI:** 10.1186/1471-2202-8-36

**Published:** 2007-05-25

**Authors:** Roberta Donato, Erik A Miljan, Susan J Hines, Sihem Aouabdi, Kenneth Pollock, Sara Patel, Frances A Edwards, John D Sinden

**Affiliations:** 1 Department of Physiology, University College London, Gower Street, London, WC1E 6BT, UK; 2 ReNeuron Limited, 10 Nugent Road, Surrey Research Park, Guildford, Surrey, GU2 7AF, UK

## Abstract

**Background:**

Neural stem cells (NSCs) are powerful research tools for the design and discovery of new approaches to neurodegenerative disease. Overexpression of the myc family transcription factors in human primary cells from developing cortex and mesencephalon has produced two stable multipotential NSC lines (ReNcell VM and CX) that can be continuously expanded in monolayer culture.

**Results:**

In the undifferentiated state, both ReNcell VM and CX are nestin positive and have resting membrane potentials of around -60 mV but do not display any voltage-activated conductances. As initially hypothesized, using standard methods (stdD) for differentiation, both cell lines can form neurons, astrocytes and oligodendrocytes according to immunohistological characteristics. However it became clear that this was not true for electrophysiological features which designate neurons, such as the firing of action potentials. We have thus developed a new differentiation protocol, designated 'pre-aggregation differentiation' (preD) which appears to favor development of electrophysiologically functional neurons and to lead to an increase in dopaminergic neurons in the ReNcell VM line. In contrast, the protocol used had little effect on the differentiation of ReNcell CX in which dopaminergic differentiation was not observed. Moreover, after a week of differentiation with the preD protocol, 100% of ReNcell VM featured TTX-sensitive Na^+^-channels and fired action potentials, compared to 25% after stdD. Currents via other voltage-gated channels did not appear to depend on the differentiation protocol. ReNcell CX did not display the same electrophysiological properties as the VM line, generating voltage-dependant K^+ ^currents but no Na^+ ^currents or action potentials under either stdD or preD differentiation.

**Conclusion:**

These data demonstrate that overexpression of myc in NSCs can be used to generate electrophysiologically active neurons in culture. Development of a functional neuronal phenotype may be dependent on parameters of isolation and differentiation of the cell lines, indicating that not all human NSCs are functionally equivalent.

## Background

Over the last decade, neural stem cells have become a major focus of interest in the development of therapies for a range of central nervous system disorders. Parkinson's disease has been perhaps the most directly pursued due to the focal and relatively well understood nature of the degeneration (for review see [1,2]). However, other presently incurable conditions, such as Huntington's and Alzheimer's diseases, also represent potential targets for such therapies (for review see [3]).

Previous research has used a wide range of neural stem cells (NSCs) from adult, fetal and embryonic origin, but mostly from rodent sources. In the case of human cells, the capacity to grow these cells while maintaining a stable phenotype across passages is limited. Thus, the development of immortal cell lines to provide suitable cells in large enough numbers as and when required is an important aim. The cells of choice for this purpose are human neural stem cells (hNSCs), which have two defining properties: self-renewal and multipotentiality. This capacity of NSCs to divide in culture and their ability to differentiate into multiple neuronal cell types makes them attractive as both a therapy for treating neurological disease and as powerful tools in the research laboratory.

Whilst a variety of rodent stem cell lines have been differentiated and transplanted into various animal models (for review see [4]) only a few earlier studies have used human embryonic or fetal stem cells and demonstrate truly functional differentiation into neurons. Functional testing is essential as it has been shown that immunological characterisation of neuronal markers does not necessarily reflect functionality [5]. Perhaps the most thorough study is that of Piper et al. who demonstrated differentiation of fetal-derived NSCs into neurons which they showed to have a variety of ligand and voltage-gated channels [6]. However, they were not fully functional as the density of voltage-dependent sodium channels was not sufficient to allow action potential generation. Another study, using cultures of human fetal brain tissue, found similar results [7]. In serum free conditions embryonic stem cells were differentiated into tyrosine hydroxylase (TH) containing cells which were demonstrated to release dopamine and have some of the electrical properties of neurons but again no action potentials were recorded. These cells were shown to survive *in vivo *in 6-hydroxydopamine lesioned rat brains [8]. Glutamatergic and GABAergic neurons have also been differentiated from human fetal forebrain under defined conditions [9]. These researchers studies clearly demonstrated ligand-gated responses, although the ability to fire action potentials was not discussed. In contrast, Perrier et al. directed embryonic stem cells to become dopaminergic neurons capable of firing action potentials using feeder layer co-cultures [10]. Another successful method was developed by Wu et al. in which pretreatment of fetal hNSCs with a mixture of fibroblast growth factor, heparin and laminin, followed by several days of differentiation, produced cholinergic neurons which reliably fired action potentials [11].

There have thus been very few reports of fully functional neurons derived from human embryonic or fetal derived stem cells. Moreover a further limitation for using many fetal-derived hNSCs has been their mortality when grown in culture [12]. For example, the cell viability in the study of Piper et al. was limited at 5 passages before differentiation [6]. Recently the life spans of human fetal-derived neural stem cells in culture have been enhanced by improvements in growth conditions [13]. However, immortalization using the myc transcription factor has proven highly effective at extending the normal life span of human NSCs *in vitro *and maintaining a stable genotype and phenotype (for reviews see [14,15]). Long term cell expansion with associated karyotype stability is a feature of myc immortalization [16]. Traditionally thought of as a proto-oncogene, it has been recently reported that myc may be a 'stemness' gene driving rapid proliferation yet maintain multipotentiality in stem cells [17].

The aim of this study was thus to develop human neuronal stem cells which can be cultured, long-term, in a proliferative undifferentiated state so that they can be expanded as needed and then differentiated into appropriate neurons. While these cell lines themselves are purely for research and could not be used in human therapy, this study paves the way for development of therapeutic stem cell lines using similar technology. We hypothesized that, using immortalized stem cell lines, cells prevented from differentiation by the presence of growth factors would lack neuronal phenotype [18,19], both when tested for morphological markers and electrophysiological criteria. In contrast, after removal of growth factors, differentiation would occur as expected. Neurons would be detectable using morphological markers as shown previously in many preparations, but this would also be accompanied by electrophysiological maturation with the development of voltage-gated channels, allowing generation of action potentials. To this end we used two immortalized stem cell lines from two anatomically distinct brain regions: midbrain (VM) and cortex (CX), and developed a protocol to maximise differentiation of the midbrain-derived cell line into dopaminergic neurons as identified by immunocytochemical markers. Both the cell lines used for this study were immortalized with the myc oncogene: ReNcell VM with v-myc while ReNcell CX was immortalized with c-myc. Moreover, ReNcell VM originated from a bulk-derived culture and ReNcell CX from a clonal cell line.

## Results

### Phenotype and function of undifferentiated ReNcell VM and CX

**Figure 1:**
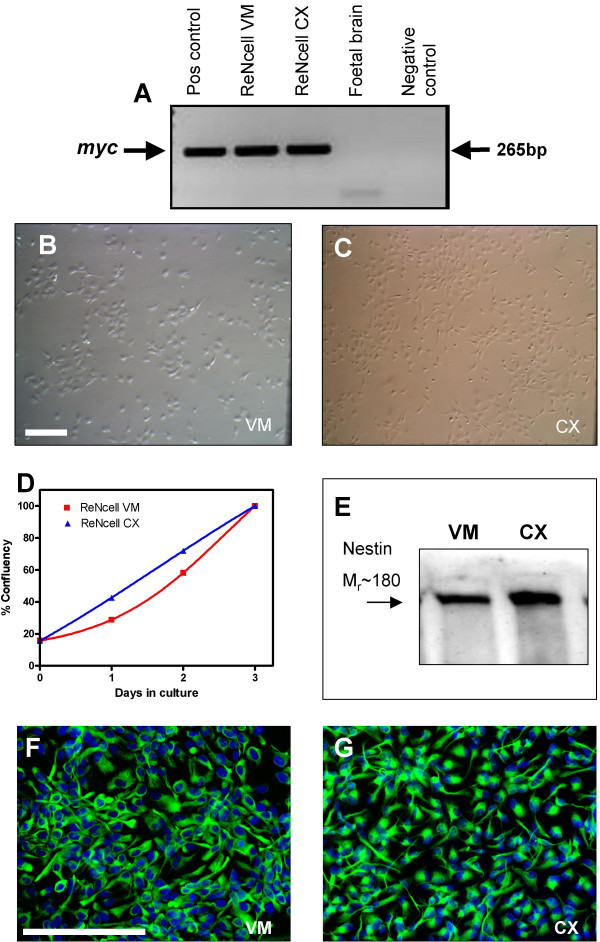
**Characterization of growth, morphology and nestin in undifferentiated cells in culture**. Cell lines are confirmed to express the myc transgene as shown by RT-PCR (A). Images of ReNcell VM (B) and CX (C) as monolayer cultures. D) Growth of cells within a passage showing cells plated at ~5000 cells/cm^2 ^achieve confluency within 3 days in culture with equivalent growth rates as measured using CyQuant fluorescent assay. ReNcell lines are positive for the neural stem cell marker nestin by western blot (E) and immunocytochemistry in green on ReNcell VM (F) and CX (G) with Hoechst nuclear counter stain in blue. (B, C) and (F, G) compare the two cell lines, scale bar: 100 μm.

**Figure 2:**
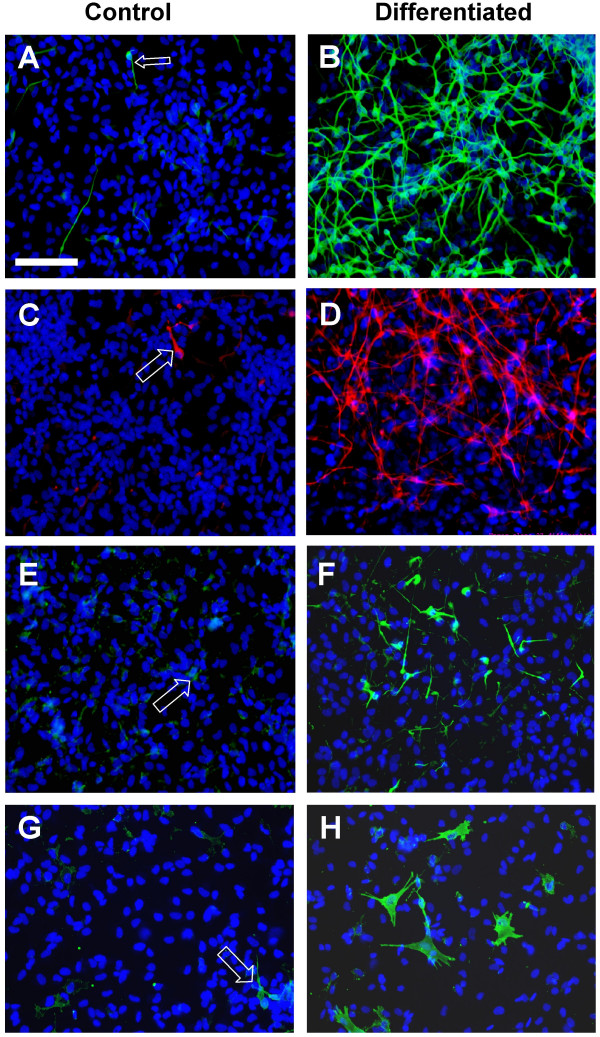
**Multipotentiality of ReNcell CX using standard differentiation assay showing neuronal, astrocytic and oligodendrocyte progeny upregulated in the differentiation state**. A-H) Representative images of control and differentiated cells showing βIII-tubulin in green (A, B); GFAP in red (C, D); O1 (E, F) and Gal C in green (G, H) with Hoechst nuclear counterstain in blue. Arrows highlight spontaneous differentiated cells in the control-undifferentiated cultures. Scale bar 100 μm for all images. Similar labelling was observed in ReNcell VM (data not shown).

ReNcell VM and CX were confirmed to carry the v-myc and c-myc transgene respectively by RT-PCR (Figure 1A). G-banded karyotype analysis of the cell lines showed a normal human male diploid (46, XY) karyotype on four independent occasions. Tested on multiple passages (26 and 36 for ReNcell VM; 3 and 30 for ReNcell CX) a normal karyotype was consistently observed, supporting the role of myc in generating karyotypically stable cell lines. Under routine growth conditions both ReNcell VM and CX displayed an immature neural morphology (Figure 1B, C): small polygonal cells with few processes that developed into a tight cobble-stone pattern when confluent. For practical reasons experiments reported here were carried out on cultures between passage 15 and 30. ReNcell CX displayed a higher degree of migration within the culture dish with a more spiny-shaped morphology than ReNcell VM. Both cell lines showed similar population doubling times of around 30 h in peak log-phase growth though the growth of the CX line was more linear (Figure 1D). Both undifferentiated cell lines expanded as islands of cells and show a positive signal for the neural stem cell marker nestin [20] as shown by western blotting (Figure 1E). This was confirmed by immunocytochemistry, with 100% of cells stained positively for nestin (Figure 1F, G). Both undifferentiated cell populations showed rare examples of spontaneously differentiated cells that were βIII-tubulin (neuronal), GFAP (astrocytic) or O1/Gal C (oligodendrocyte) positive (representative images of ReNcell CX shown in Figure 2A, C, E, G).

**Figure 3:**
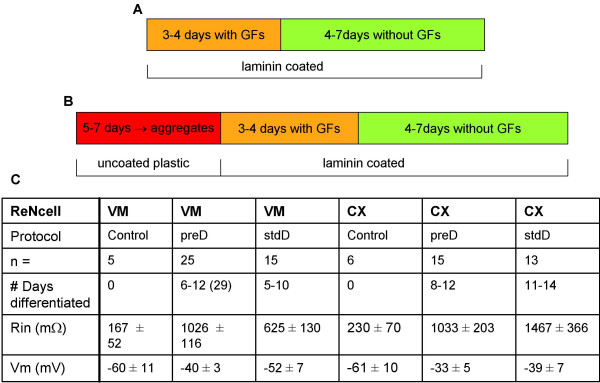
**Differentiation protocols used and the passive properties of different cell types under the different protocols**. Schematic diagrams of stdD (A) and preD (B) differentiation protocols. The passive properties of undifferentiated (control) and differentiated (stdD and preD) ReNcell VM and CX are compared (C).

**Figure 4:**
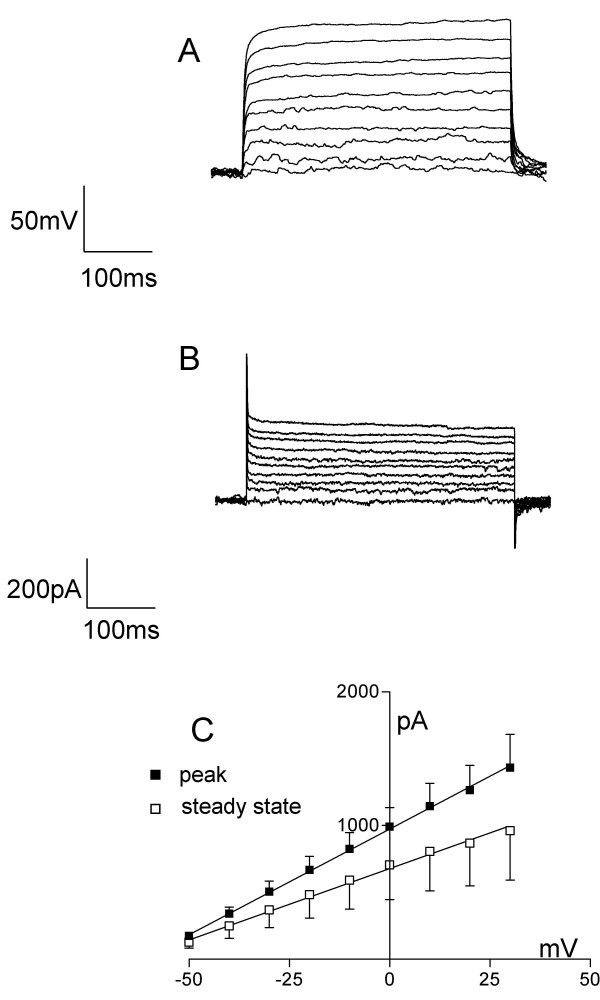
**Current-voltage relationships in undifferentiated ReNcell VM**. A) Example of voltage responses from a resting potential of ~-80 mV to current steps of increasing amplitude in a cell (50 pA steps from 50 pA to 500 pA, 400 ms duration). B) Current responses to voltage steps of 10 mV, from the holding potential of -60 mV to +30 mV, in the same cell shown in A). C) Average voltage-current relationship for the peak (solid squares) and the steady (open squares) current in undifferentiated ReNcell VM (n = 4). The lines represent linear regressions of the two data sets with r^2 ^> 0.99 in both cases.

Whole cell patch clamp techniques were used to investigate the electrophysiological properties of the cells. Current-voltage relationships were recorded in undifferentiated cells at 3–4 days after passage onto coverslips. For ReNcell VM, data were obtained from five cells from five different coverslips. Resting membrane potential in these cells was -60 ± 11 mV and input resistance 167 ± 52 MΩ (mean ± SE, n = 5). ReNcell CX cells displayed similar properties with a resting membrane potential of -61 ± 10 mV and input resistance of 230 ± 70 MΩ, (n = 6, see Figure 3C). Under current clamp, only an Ohmic change in membrane potential was seen in either cell line in response to stepping up current in 50 pA increments (for ReNcell VM see Figure 4A). Similarly, under voltage clamp, no voltage-dependent changes in current were evoked over a range of holding potentials, -60 to +30 mV in either ReNcell VM or CX (Figure 4B). Indeed, both the peak and steady state current voltage relationships were linear throughout the voltage range tested (Figure 4C), indicating the absence of voltage-activated conductances.

### Maturation of ReNcell VM and CX phenotype following differentiation

Initially a standard monolayer differentiation (stdD) protocol in laminin coated wells was used with growth factors simply being omitted from the medium and differentiation continuing under the same culture conditions as had been used for growth of the undifferentiated cells (Figure 3A). Mitogen withdrawal is considered to be the simplest method to induce differentiation of neural stem cells [19] and is therefore termed "standard differentiation" in our laboratory. Under these conditions morphological and phenotypic changes in both cell lines were noted following initiation of differentiation. Within 4 days both cell lines acquired clearly defined, rounded cell bodies with extensive neurite outgrowths (ReNcell CX shown in Figure 2B). Using immunocytochemistry following stdD, cells from all three neuronal lineages – neurons, astrocytes and oligodendrocytes – were positively identified (ReNcell CX shown in Figure 2D, F, H. Similar results were found for ReNcell VM, results not shown).

Neural stem cells have the ability to grow as neurospheres [21,22]. We investigated the effect of selecting cells from neurospheres before plating onto laminin-coated wells and following the standard procedure. This was achieved by plating undifferentiated cells onto uncoated plastic to which the cell adhere weakly and migration does not occur. After leaving the cells untouched for 5–7 days in medium plus growth factors, many cells died but a proportion of the cells expanded into neurosphere-like structures approximately 100–200 μm in diameter. When such spheres were transferred to laminin-coated dishes and then kept in an undifferentiated state for a further 3–4 days before removal of growth factors, the cells from the sphere migrated onto the substrate during the growth phase and formed a confluent monolayer prior to initiation of differentiation.

**Figure 5:**
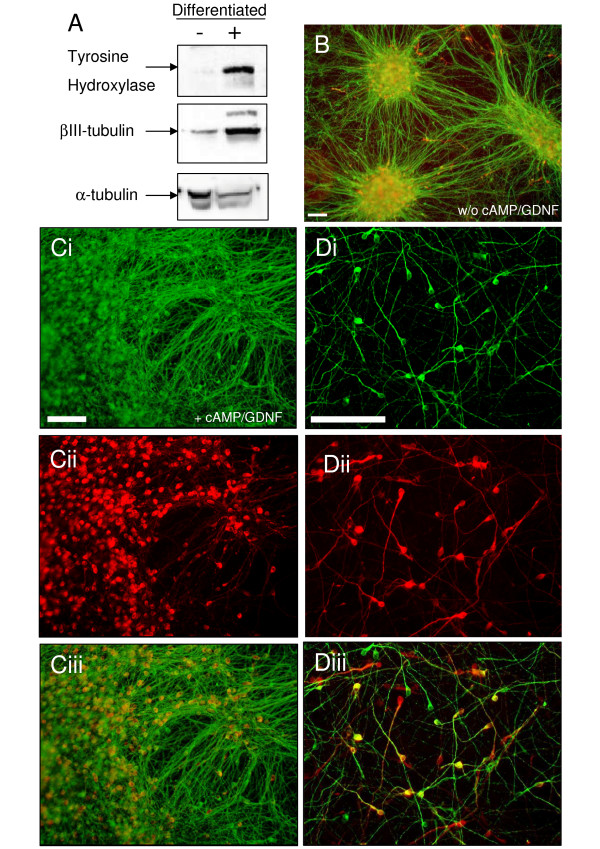
**Differentiation of ReNcell VM into a dopaminergic neural phenotype**. A) Using the preD protocol there is a significant increase in TH and βIII-tubulin protein expression as assayed by western blot (- undifferentiated, + differentiated with α-tubulin as a loading control). B) Following preD (without cAMP/GDNF), the cells are gathered in clusters which are strongly labelled with βIII-tubulin (green). TH (red) positive neurons are also evident but the clustering makes colocalization difficult to differentiate (data not shown). Ci, ii, iii) ReNcell VM preD in the presence of cAMP/GDNF shows a more even distribution of neuronal differentiation positive for βIII-tubulin (Ci, green) and TH (Cii, red), with the overlay showing co-localization of the two antigens (Ciii). Di, ii, iii). A higher resolution image, under the same conditions as C, show individual neurons stained with βIII-tubulin (Di, green) and TH (Dii, red), with the overlay showing co-localization (Diii). Scale bar: 100 μm.

Clearly, when growth factors were removed (as for the standard procedure) both ReNcell CX and VM cell lines differentiated and showed strong labeling for the neuronal marker βIII-tubulin (Figure 5A, B, Cii, Dii). However, differences between the two stem cell lines became apparent at this stage of the preD protocol. Cells from the ReNcell CX line spread across the dish behaving similarly to those under the standard protocol described above. In contrast, the cells from the ReNcell VM tended to bunch into dense patches. This could be prevented by adding cAMP and GDNF to the medium during the differentiation phase (compare Figures 5B &5Ci). Subsequently this enriched medium was used for these studies for both cell lines. It seems likely that the lack of foci in the enriched medium may arise from an increase in cell motility, potentially enhanced by the addition of GDNF [23], but this was not further studied.

### Development of dopaminergic phenotype in ReNcell VM neurons but not in ReNcell CX neurons

We were particularly interested in finding conditions in which these cell lines might differentiate into dopaminergic neurons. However, staining for the dopaminergic marker TH was not observed in the differentiated ReNcell CX neurons (data not shown). In contrast, after differentiation, strong TH expression was shown in the ReNcell VM neurons both by Western blot and immunocytochemical analysis (colabelling of TH and βIII-tubulin, Figure 5). Interestingly, the pre-aggregation differentiation (preD, Figure 3B) protocol (with the addition of cAMP and GDNF) seemed to enhance the dopaminergic phenotype of the ReNcell VM line. Although TH staining alone does not provide conclusive evidence that these are indeed dopaminergic neurons, it is sufficient for the purpose of monitoring and optimization of the differentiation protocols.

### Electrophysiological characteristics of differentiated ReNcells using the preD protocol

**Figure 6:**
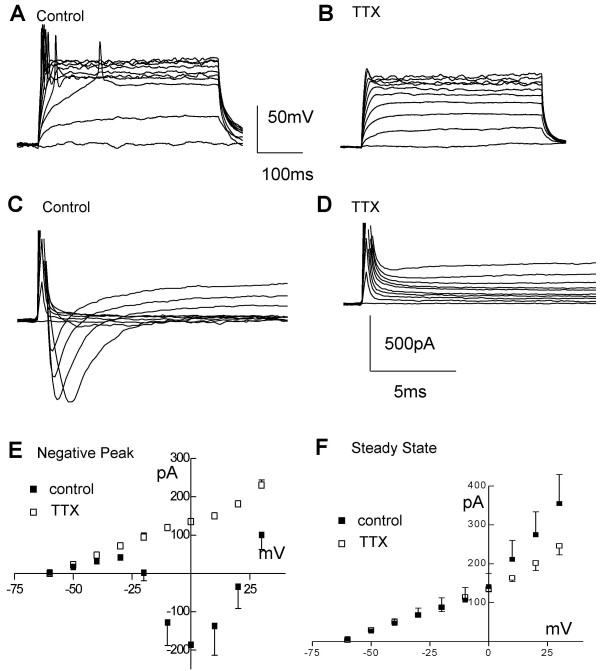
**ReNcell VM current-voltage responses following preD**. A, B) Example of firing elicited by stepping the current command in 20 pA steps (400 ms duration) from a resting potential of ~-75 mV in ReNcell VM 10 days after differentiation was started in control solution (A) and after adding 0.6 μM TTX (B). C, D) Same cell as in (A, B) responding with inward currents to incremental voltage steps (10 mV, from -60 mV to + 30 mV, 400 ms duration), before (C) and after (D) adding 0.6 μM TTX to the control solution. E) Average inward current-voltage relationships in control differentiated (solid squares) and after adding 0.6 μM TTX (open squares) and F) average steady-state current-voltage relationship for control (solid squares) and TTX treated (open squares).

In contrast to the undifferentiated cells described above, differentiated ReNcell VM consistently displayed active currents. Recordings were made during the second week after differentiation. When current steps were injected at this stage, action potentials were elicited in 8/8 cells tested (Table 1; Figure 6A). An additional 2/2 cells tested after 29 days of differentiation also featured action potentials. While we did not concentrate on testing these later time points, this indicates that at least in some cases long-term survival of the neurons is possible. If tested earlier, at 6 days after differentiation, the occurrence of action potentials was less consistent (9/15 cells) and, unlike at later stages, may have been dependent on plating density (Table 1). ReNcell VM action potentials were consistently blocked in the presence of 0.6 μM tetrodotoxin (TTX). In voltage clamp experiments the current responses to voltage steps activated a fast inward current, which was abolished in the presence of TTX (Figure 6C, D). Figure 6E and 6F show the average and steady state peak inward current evoked by stepping the command voltage from -60 to +30 mV in 10 mV increments (n = 14). The fast inward current was activated at voltages positive to -20 mV and reversed at voltage positive to +20 mV. The fact that the current was abolished in the presence of 0.6 μM TTX indicated that it was mediated by the activation of the type of Na^2+^-channels which underlies the generation of action potentials. The average steady-state current-voltage relationship was also non-linear (Figure 6F) suggesting that other voltage-gated channels were also activated.

**Table 1:** Development of action potentials in preaggregated VM ReNcells is independent of plating density

**Plating Density**	**Cells with APs/total tested**
**6 days differentiation**	
1:1	1/2
1:2	2/7
1:4	6/6
**> 8 days differentiation**	
1:2	3/3
1:4	4/4
1:8	3/3

### Other voltage-gated channels

While we have not studied other channels in detail, it is clear that some other voltage-gated channels are present. An important contributing conductance is probably the potassium conductance, I_A_. (See Methods section for the protocol for isolation of I_A_). Current consistent with I_A _was present in all cells tested though we cannot discount a possible contribution from Ca^2+ ^channels or other channels differentially activated by the two protocols (data not shown).

### Comparison between preD and stdD protocols

The preD protocol was used for the above experiments because of the observation that it enhanced the proportion of TH-positive neurons among the differentiated ReNcell VM neurons. To investigate whether the preD protocol affected the development of action potentials, we also studied ReNcell VM using the original stdD protocol. StdD cultures were studied in the same time frame as for the preD ReNcell VM, 7–14 days after differentiation. In these conditions, cells displayed a resting membrane potential of -52 ± 7 mV and an input resistance of 625 ± 130 MΩ (n = 15). However, only in 25% of cases (4/15) could Na^+ ^conductances be activated by stepping the voltage towards positive values. Even for those cells with inward currents the Na^+ ^currents were on average considerably smaller and more varied than for the preD protocol (data not shown). Unlike for sodium currents, the amplitude and voltage dependency of I_A _in differentiated ReNcell VM was independent of the protocol used.

### Development of voltage-gated currents in ReNcell VM but not CX

**Figure 7:**
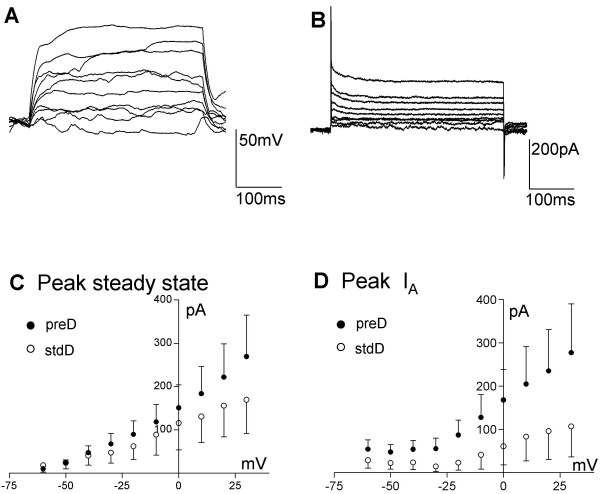
**Electrophysiological properties of ReNcell CX**. A, B) Example of ReNcell CX 12 days after differentiation was started using the preD protocol, responding to current (A; from ~-80 mV) or voltage (B) steps (10 pA or mV increments, 400 ms). C) Average voltage-current relationship for the peak current elicited by stepping the voltage from -60 to + 30 mV in 10 mV increments for the ReNcell CX with the preD (solid circles, n = 9) or the stdD (open circles, n = 7) protocols. D) Average I_A _peak current for the ReNcell CX differentiated using preD (filled circles, n = 9) or the stdD (open circles, n = 7) protocols. Despite the apparent trend there is no significant difference between these groups due to the large scatter in the data.

Having observed that, although not completely dependent on preD, the development of Na^+ ^currents was substantially enhanced by the preD compared to the stdD, we then checked both protocols in ReNcell CX. ReNcell CX cells studied over the second week after preD had a resting membrane potential of -33 ± 5 mV and an input resistance of 1033 ± 203 MΩ (n = 15). No action potentials or inward currents could be generated in any of the cells tested (0/12) in response to injection of current or voltage in steps of increasing positive amplitude (from a starting potential of ~-80 mV, Figure 7A, B). Like ReNcell VM cells, ReNcell CX displayed a non-linear current-voltage relationship (Figure 7C, n = 9), suggesting the presence of voltage-activated conductances.

We have restricted our analysis of other voltage-gated channels to I_A_. We find that the presence of I_A _was independent of cell type or protocol being similar to that seen in ReNcell VM (Figure 7D). The passive properties of ReNcell CX are compared to those of ReNcell VM under different conditions in Figure 3C. Overall, in contrast to the ReNcell VM neurons, the use of the preD protocol did not show any measurable effects on ReNcell CX neurons.

## Discussion

We have developed immortalized human fetal neural stem cell lines from the cortex (CX) and from the ventral mesencephalon (VM) regions of the brain and tested their differentiation potential and functional capacity under different culture conditions. These two brain areas were chosen as they are of particular interest as research models of cellular processes occurring during the development of degenerative diseases such as Huntington's, Alzheimer's and Parkinson's diseases. Our initial hypothesis that morphological differentiation of the stem cells into neurons would also be reflected in their electrophysiological characteristics was not supported by the data.

When comparing the two stem cell lines under the same culture conditions, undifferentiated ReNcell VM and CX displayed very similar phenotypes. Both cell lines in the undifferentiated state were indistinguishable in their nestin expression, as well as their electrophysiological properties. Moreover, on differentiation immunohistochemical characteristics were also similar in that both cell lines showed a similar level of neuronal development as judged from staining for βIII-tubulin though only the mesencephalic stem cells showed significant positive staining for TH, a marker for dopaminergic neurons. However, despite the immunological evidence of neuronal development in both cell lines, functional evidence for neuronal differentiation – the ability to generate action potentials – was only observed in the ReNcell VM neurons. ReNcell CX cells cultured under identical conditions did not develop this physiological phenotype.

This aspect of neuronal function was particularly enhanced in ReNcell VM by allowing the cells to form neurospheres before plating and subsequent removal of growth factors to allow differentiation. Interestingly, although the capacity to form neurospheres was also evident in the cortical stem cells, this predifferentiation step made no apparent difference to the subsequent differentiation of ReNcell CX cells.

It has previously been proposed that the differences seen between electrophysiology studies of NSCs are due to variations in culture conditions [7]. ReNcell VM and CX were both maintained long-term in identical culture conditions and although the same differentiation and recording protocols were used, the two lines gave different functional outcomes. This suggests that the two cell lines are either intrinsically different or neuronal maturation is affected by differences in derivation of the cell lines. It is possible that similar outcomes may in the future be achieved by designing differentiation protocols specific for ReNcell CX to generate functional neurons. There are, however, significant differences and this may relate to how the cell lines were actually derived, either in terms of: 1) the brain area from which they are derived; 2) the derivation of ReNcell CX from a clonal cell line versus ReNcell VM from a bulk derived culture; or 3) the use of v-myc versus c-myc for immortalization of the lines.

It is unlikely that the area of the brain from which the cells are derived results in this difference as it is clear that stem cells must exist in fetal cortical tissue which can differentiate into fully functional neurons [11]. However, whether the cells are clonal or derived from bulk tissue may be a factor leading to the variability in the results observed between the two cell lines. The second difference seems a more likely source of the final outcomes observed. It is possible that the myc integration site within the genome of the clonal ReNcell CX line has disrupted a gene expression pattern required for functional neural differentiation. Alternatively, in the case of development of the VM line from bulk tissue, a variety of neural stem cells with varying degrees of commitment remain in the culture while the CX cells derived from a single initial cell would only represent one subtype of stem cell. Thus, contrary to the assumption that neural stem cells derived from fetal tissue are all equally multipotent, this may suggest that within any tissue, a variety of stem cells exists each with its own committed limits of differentiation. Each type may be able to proliferate indefinitely and differentiate into immunologically identifiable neurons, astrocytes and oligodendrocytes. They would thus be defined as neural stem cells [21,24] but the eventual potential or requirements for functional differentiation into specific cell types may vary within the population. Neurosphere formation may select for a particular cell subtype which can compete best under these conditions which can subsequently differentiate into fully functional neurons, including dopaminergic neurons. The CX line, being derived from a single cell, would not have such variation and thus selecting neurospheres would make no difference as the cells would all be identical. Interestingly, only one previous study has succeeded in differentiating similarly derived human fetal stem cells to become functional neurons which were confirmed to fire action potentials [11]. Rather than being clonal, these cells were derived from cortical bulk tissue thus further supporting our suggestion that it is the subsequent preparation of the cells and not their origin which is important in the differences seen.

We cannot discount the possibility that these outcomes originate from the third difference listed above – the use of different proto-oncogenes for immortalization. ReNcell VM was immortalized using v-myc and ReNcell CX with c-myc proto-oncogenes. c-Myc is described as the human homolog of avian v-myc [25]. Although c-myc is larger in size, they are considered to be homologous because they contain the same transforming domains [26] with the same subcellular localization to the nucleus [27]. v-Myc may be a more potent immortalization agent than c-myc. This has been attributed to mutations in the v-myc gene which enhance the transformation ability of myc [28]; however, the presence of v-myc polymorphisms has not been investigated in ReNcell VM. Moreover, during differentiation of human NSCs, myc transgene expression was shown to be down-regulated [16]. The presence of v-myc cannot, however, be the primary determining factor because Cho et al. did not observe action potentials in v-myc derived human NSCs [7].

## Conclusion

We conclude that: 1) immunocytochemical characterization is not sufficient to identify the functional maturation of different cell types and 2) differences in culturing conditions might enhance the property of generating functional neurons, but the property itself seems to be intrinsic to a specific cell line which may arise from the method used to prepare the cell line. Further studies are required to elucidate the determining factors for human fetal derived neural stem cells to be maturated into functional neurons.

## Methods

### Cell line derivation

The procedure to derive cell lines is similar to that recently reported [29]. The ReNcell VM line was derived from ten-week gestation fetal midbrain tissue that was obtained from Kings College Hospital, London, whereas the ReNcell CX line was derived from a 14-week gestation fetal cortex obtained from Advanced Bioscience Resources (Alameda CA, USA), both following normal terminations and in accordance with nationally (UK and/or USA) approved ethical and legal guidelines. The tissues were collected in ice-cold HBSS without Ca^2+^/Mg^2+ ^(Gibco), containing 1 mM N-acetyl cysteine (Sigma). The appropriate brain region was isolated and cleaned under a dissection microscope. Single cells were isolated by chopping the tissue with a scalpel and then incubating in 0.25% w/v trypsin (BioWhittaker) in DMEM:F12 (Gibco) containing 25 U/ml Benzonase (Merck) for 15 min at 37°C. Following the dissociation, twice the volume of trypsin inhibitor solution (0.55 mg/ml soybean trypsin inhibitor, Sigma, dissolved in DMEM:F12, containing 1% human serum albumin (Grifols) and 25 U/ml Benzonase) was added. Cells were centrifuged at 800 g for 5 min, then resuspended in serum free human media comprising DMEM:F12 supplemented with human serum albumin (0.03%), human transferrin (100 μg/ml, Sigma), putrescine dihydrochloride (16.2 μg/ml, Sigma), human insulin (5 μg/ml, Sigma), L-thyroxine (T4, 400 ng/ml, Sigma), tri-iodo-thyronine (T3, 337 ng/ml, Sigma), progesterone (60 ng/ml, Sigma), L-Glutamine (2 mM, Gibco), sodium selenite (selenium, 40 ng/ml, Sigma), heparin, sodium salt (10 Units/ml, Sigma), corticosterone (40 ng/ml, Sigma) and Gentamycin (50 ug/ml, Gibco) in the presence of bFGF (10 ng/ml, Invitrogen) and EGF(20 ng/ml, Sigma). Dissociated cells from the two different brain regions were plated on laminin coated flasks (Invitrogen, coated at 20 μg/ml in DMEM:F12 for 1 hr at 37°C followed by one wash with warm DMEM:F12) at a concentration of 5 × 10^4 ^cells/cm^2 ^and maintained at 37°C in a humidified atmosphere of 95% air/5% CO_2_. MMLV type retrovirus encoding the v- or c- myc genes was generated using the TEFLY-A (amphotropic) virus packaging cell line as previously described [30]. Midbrain derived cells were then infected for 8 h with fresh media containing retrovirus carrying the v-myc gene in the presence of 4 μg/ml polybrene, whereas the cortex derived cells were infected under the same conditions except c-myc containing retrovirus was used, both driven under the CMV promoter. Three successive infections were carried out. Following infection, cells were placed under antibiotic selection (ReNcell VM 150 μg/ml geneticin and ReNcell CX 75 μg/ml hygromycin, both supplied by Gibco). Putting the bulk infected mid-brain culture under selection for 2–3 weeks generated the ReNcell VM line. The cortical cells were plated at low density (~2000 cells) in a 15 cm diameter tissue culture dish and put under selection for 2–3 weeks. Resistant colonies were picked using the cloning ring method (8 mm cloning ring, Sigma). A series of approximately 15 clonal cell lines were isolated (data not shown). Clone 27 was designated ReNcell CX and moved forward because of its capacity to differentiate into morphologically distinct neurons. Confirmation of myc transgene expression in both ReNcell VM and CX was confirmed by RT-PCR using the following primer sequences: (5' – 3') AGCTGGTTTAGTGAACCGTCAGATC (F); AGCAGCTCGAATTTCTTCCA (R) (Sigma). The forward primer sequence is targeted against the CMV promoter shared by both transgenes and the reverse primer against a homologous region between c-myc and v-myc with a single band product from each of 265 bp.

### Cell culture

Having established the cell lines as described, cells were routinely expanded for experimental work on laminin coated T75 cm^2 ^or T175 cm^2 ^tissue culture flasks (Nunc Easyflask) in B27 medium [DMEM:F12 containing B27 neural cell supplement mix (Gibco), L-Glutamine (2 mM, Gibco), heparin (10 Units/ml, Sigma) and Gentamicin (50 μg/ml, Gibco)] in the presence of bFGF (10 ng/ml, Invitrogen) and EGF (20 ng/ml, Sigma) – referred to as growth medium. All cells in culture were maintained at 37°C in a humidified atmosphere of 95% air/5% CO_2_. It is important not to allow the cells to become confluent (> 80% confluency) at any time. Cells were passaged every three to four days using trypsin and trypsin inhibitor solutions. Briefly, cells were rinsed with HBSS without Ca^2+^/Mg^2+ ^and then incubated in trypsin solution for 5–15 min until the cells detached. Twice the volume of trypsin inhibitor was added and the cells centrifuged at 500 g × 5 min. The cell pellet was resuspended in fresh medium and plated in a freshly laminin coated flask(s) at a density of ~10000 cells/cm^2^. For consistency and practical reasons, all experiments presented in this study were carried out on cells between passages 15 and 30. However, karyotype analyses were carried out on longer-term cultures and found to be normal.

### Cell proliferation assays

To monitor the growth rate, cells were seeded at 2,000 cells/well on laminin coated 96-well tissue culture plates (removable strip plates, Corning). Every day two strips were removed (n = 16), media aspirated, and plates stored dry at -70°C. The relative number of cells was determined using CyQuant cell proliferation assay (Molecular Probes) according to the manufacturer's instructions. Fluorescent measurements were made on a microplate reader (Tecan, Genios).

### Differentiation

Cells were passaged into laminin-coated 96-well plates (BD Biosciences) for differentiation assays. Differentiation of both cell lines was carried out using two different protocols. For standard differentiation (stdD) assays, cells were seeded at 30,000 cells/cm^2 ^in a 96-well plate (10,000 cells/well) and expanded to confluency in growth medium over a 3–4 day period. Differentiation was initiated by changing the medium to that without growth factors. Following approximately 4–7 days' differentiation, cells were fixed for immunocytochemistry. The pre-aggregation differentiation (preD) protocol was observed to enrich the dopaminergic phenotype differentiation of the ReNcell VM line. Aggregates were made by seeding 30,000 cells/cm^2 ^(10,000 cells/well) on uncoated 96-well plates in growth medium and left expanding for 7 days with no media change. At this point, the aggregates were harvested and dissociated by gentle trituration and replated on laminin-coated 96-well plates with the dissociated cells from one aggregate preparation placed in an equivalent sized well (plating density of 1:1 based on surface area). The cells were then differentiated using the stdD assay as described above. Where described, 1 mM dibutyrl-cAMP (Calbiochem) and 2 ng/ml GDNF (Peprotech) was added to the differentiation media. In some cases plating was sparser with an aggregate preparation being divided between two or more wells (plating density of 1:2; 1:3 etc).

### Immunocytochemistry

Following growth and/or differentiation on laminin-coated 96-well culture plates, the medium was removed and the cells fixed for 15 minutes in cold 4% paraformaldehyde/PBS followed by two PBS washes. Cells were permeabilised with 0.1% Triton × 100 in PBS for 15 minutes and non- specific binding was blocked with 10% normal goat serum (NGS, Vector labs) in PBS for 1 hour at room temperature. Nestin was probed using mouse monoclonal anti-nestin at 1:600 (Chemicon, MAB5326), βIII-tubulin was probed using a mouse monoclonal at 1:1000 (Sigma, UK), anti-GFAP rabbit polyclonal at 1:5000 (DAKO), and anti-tyrosine hydroxylase (TH) used at 1:250 (Chemicon, AB152). Primary antibodies were incubated overnight at room temperature. After washing twice with PBS, they were then processed with filtered Alexa dye conjugated Goat anti-Mouse 488 (1:250; Molecular Probes) or Alexa dye conjugated Goat anti-Rabbit 568 (1:2500; Molecular Probes) dissolved in 1% NGS in PBS for 1.5 hour at room temperature. Cells were washed with PBS and counterstained with 10 mM Hoechst 33342 (Sigma) for 4 minutes followed by an additional PBS wash.

### Western blot

Protein expression in cell samples from 96-well plates were analyzed by western blotting. Cells were gently washed twice for 5 minutes each time with 200 μl PBS and subsequently lysed with 50–150 μl 1X SDS-Sample buffer (15 mM Tris pH 6.8, 5% glycerol, 1% β-mercaptoethanol, 1% SDS, 0.002% bromophenol blue). The cell lysate was then heated for 5 min at 70°C prior to resolution on a 10 well × 1 mm thick SDS-PAGE (Novex) and blotted to nitrocellulose membrane (Whatman Schleicher & Schuell BioScience, BA85) using a Mini-VE blotting apparatus (Invitrogen) as per manufacturers' guidelines. Non-specific binding of the blotted membrane was blocked with 5% non-fat dried milk diluted in wash buffer (0.1% tween 20/PBS) for 1 h. The membrane was then probed with anti-nestin (ATCC, 2 μg/ml), tyrosine hydroxylase (Chemicon, AB152, 1:250), βIII-tubulin (Sigma, 1:500) or α-tubulin (Sigma, 1:1000) diluted in wash buffer for 1 hr. After three washes, the membranes were incubated in peroxidase-linked secondary antibodies (anti-mouse HRP or anti-rabbit HRP, Amersham, 1:5000) for 1 hour then washed and developed with Supersignal West Femto detection kit (Pierce). Images were captured using a Bio-Rad Fluor-S digital imaging system.

### Electrophysiology

For electrophysiology studies, cells were plated on 13 mm diameter laminin coated Thermanox (Nalge Nunc International) coverslips in the growth medium. Control cells were grown in growth media and measured following 3–4 days' growth. In general protocols for stdD and preD are followed as described above. In the case of ReNcell VM samples prepared for electrophysiology using the preD protocol, the seeding density is expressed as the ratio of dilution of the aggregates following 7 days expansion from an initial seeding density of 30000 cells/cm^2^. At specified times (days of differentiation), coverslips were placed in a recording chamber on the stage of an upright microscope (Olympus). Cells were perfused with an extracellular solution containing (in mM): 125 NaCl, 2.4 KCl, 2 CaCl_2_, 1 MgCl_2_, 26 NaHCO_3_, 1.1 NaH_2_PO_4_, 10 glucose, and bubbled with 95% O_2 _/5% CO_2_. The conventional whole cell patch clamp technique [31] was employed by using an an Axopatch 1-D patch-clamp amplifier, (Axon Instruments, Foster City, CA) both for current clamp and voltage clamp recordings. Electrodes were pulled from thick walled borosilicate glass (World Precision Instruments, Hertfordshire, UK) to a tip resistance of 4–6 MΩ (PP-83 microelectrode puller; Narishige, Tokyo, Japan) when filled with an intracellular solution containing (in mM): 130 KGluconate, 10 NaCl, 10 HEPES, 10 EGTA, 2 MgATP, and 1 MgCl_2_, pH 7.4 with NaOH and osmolality 298 mOsm with 25 glucose. Voltage and current pulse generation and data acquisition were performed with a PC running the WCP software (University of Strathclyde, Scotland). Data were sampled at 10 kHz with an A/D interface (Digidata 1200). Membrane potentials were corrected off line for the liquid junction potential, which was measured as -15 mV. Cell input resistance (R_*in*_) was estimated from the current response to small (5 mV) hyperpolarizing voltage command from the holding potential (-60 mV). The protocols for measuring Na^+ ^currents and action potentials are detailed in the figure legends. The voltage clamp protocol for testing active potassium I_A _conductance consisted of a 400 ms hyperpolarizing pulse from a holding potential of -60 to -120 mV to remove inactivation, followed by 400 ms depolarizing steps in 10 mV increments at 0.5 Hz. This protocol was then followed by a similar one but with the prepulse at +30 mV in order to inactivate the I_A _conductance. Digitally subtracting the responses from these two protocols results in an estimate of the pure I_A_current.

## Abbreviations

NSCs neural stem cells

hNSCs human neural stem cells

VM ventral mesencephalon

CX cortex

stdD standard differentiation protocol

preD preaggregate differentiation protocol

TH tyrosine hydroxylase

TTX tetrodotoxin

GFs growth factors bFGF and EGF

## Authors' contributions

EM derived the two cell lines used in this study, with the support of ReNeuron staff, and developed the pre differentiation protocol, in addition to preparing the samples for electrophysiology. RD carried out electrophysiological experiments and analysis. SH and SA prepared and analyzed the cells by immunocytochemistry. FE, KP, SP and JS conceived the study, participated in its design, coordinated and helped draft the manuscript. All authors read and approved the final manuscript. The study was funded by ReNeuron.
